# A 14-Item Mediterranean Diet Assessment Tool and Obesity Indexes among High-Risk Subjects: The PREDIMED Trial

**DOI:** 10.1371/journal.pone.0043134

**Published:** 2012-08-14

**Authors:** Miguel Angel Martínez-González, Ana García-Arellano, Estefanía Toledo, Jordi Salas-Salvadó, Pilar Buil-Cosiales, Dolores Corella, Maria Isabel Covas, Helmut Schröder, Fernando Arós, Enrique Gómez-Gracia, Miquel Fiol, Valentina Ruiz-Gutiérrez, José Lapetra, Rosa Maria Lamuela-Raventos, Lluís Serra-Majem, Xavier Pintó, Miguel Angel Muñoz, Julia Wärnberg, Emilio Ros, Ramón Estruch, for the PREDIMED Study Investigators

**Affiliations:** 1 PREDIMED (“Prevención con dieta mediterránea”) Network (RD 06/0045), Instituto de Salud Carlos III (ISCIII), Spanish Government, Madrid, Spain; 2 Department of Preventive Medicine and Public Health, University of Navarra, Pamplona, Spain; 3 CIBER (Center for Biomedical Network Research) Fisiopatología de la Obesidad y Nutrición (CIBERobn) Instituto de Salud Carlos III (ISCIII), Spanish Government, Madrid, Spain; 4 Human Nutrition Department, IISPV, Universitat Rovira i Virgili, Reus, Spain; 5 Department of Preventive Medicine, University of Valencia, Valencia, Spain; 6 Cardiovascular and Nutrition Research Group, Institut de Recerca Hospital del Mar, Barcelona, Spain; 7 Department of Cardiology, University Hospital of Alava, Vitoria, Spain; 8 Department of Preventive Medicine, University of Malaga, Malaga, Spain; 9 Institute of Health Sciences IUNICS, University of Balearic Islands and Hospital Son Espases, Palma de Mallorca, Spain; 10 Instituto de la Grasa, Consejo Superior de Investigaciones Cientificas, Sevilla, Spain; 11 Department of Family Medicine, Primary Care Division of Sevilla, San Pablo Health Center, Sevilla, Spain; 12 Department of Nutrition and Food Science, School of Pharmacy, XaRTA, INSA, University of Barcelona, Barcelona, Spain; 13 Department of Clinical Sciences, University of Las Palmas de Gran Canaria, Las Palmas, Spain; 14 Lipids and Vascular Risk Unit, Internal Medicine, Hospital Universitario de Bellvitge, Hospitalet de Llobregat, Barcelona, Spain; 15 Primary Care Division, Catalonian Institute of Health, Barcelona, Spain; 16 Lipid Clinic, Department of Endocrinology and Nutrition Institut d’Investigacions Biomediques August Pi Sunyer (IDIBAPS), Hospital Clinic, University of Barcelona, Barcelona, Spain; 17 Department of Internal Medicine, Institut d’Investigacions Biomediques August Pi Sunyer (IDIBAPS), Hospital Clinic, University of Barcelona, Barcelona, Spain; Universidad Autonoma Madrid, Spain

## Abstract

**Objective:**

Independently of total caloric intake, a better quality of the diet (for example, conformity to the Mediterranean diet) is associated with lower obesity risk. It is unclear whether a brief dietary assessment tool, instead of full-length comprehensive methods, can also capture this association. In addition to reduced costs, a brief tool has the interesting advantage of allowing immediate feedback to participants in interventional studies. Another relevant question is which individual items of such a brief tool are responsible for this association. We examined these associations using a 14-item tool of adherence to the Mediterranean diet as exposure and body mass index, waist circumference and waist-to-height ratio (WHtR) as outcomes.

**Design:**

Cross-sectional assessment of all participants in the “PREvención con DIeta MEDiterránea” (PREDIMED) trial.

**Subjects:**

7,447 participants (55–80 years, 57% women) free of cardiovascular disease, but with either type 2 diabetes or ≥3 cardiovascular risk factors. Trained dietitians used both a validated 14-item questionnaire and a full-length validated 137-item food frequency questionnaire to assess dietary habits. Trained nurses measured weight, height and waist circumference.

**Results:**

Strong inverse linear associations between the 14-item tool and all adiposity indexes were found. For a two-point increment in the 14-item score, the multivariable-adjusted differences in WHtR were −0.0066 (95% confidence interval, –0.0088 to −0.0049) for women and –0.0059 (–0.0079 to –0.0038) for men. The multivariable-adjusted odds ratio for a WHtR>0.6 in participants scoring ≥10 points versus ≤7 points was 0.68 (0.57 to 0.80) for women and 0.66 (0.54 to 0.80) for men. High consumption of nuts and *low* consumption of sweetened/carbonated beverages presented the strongest inverse associations with abdominal obesity.

**Conclusions:**

A brief 14-item tool was able to capture a strong monotonic inverse association between adherence to a good quality dietary pattern (Mediterranean diet) and obesity indexes in a population of adults at high cardiovascular risk.

## Introduction

The overall quality of a dietary pattern appears to affect adiposity and the risk of obesity to a greater extent than relative macronutrient quantity [1]–[3]. However, only some prospective studies have investigated the association of diet quality scores with obesity risk [1], [4]–[11]. Most of them found that an overall food pattern in line with the traditional Mediterranean diet was inversely associated with obesity risk or weight gain [1], [4], [6], [10]–[11]. This “Mediterranean” dietary pattern is typically based on whole or minimally processed foods and incorporates most of the protective factors (fruits and vegetables, legumes, whole grains, dietary fiber, fish, vegetable protein and vegetable fat from olive oil) but few of the adverse dietary factors (fast food, sugar-sweetened beverages, refined grain products, energy density, and partially hydrogenated or trans-fat) for obesity [2]. The inverse association between the Mediterranean diet and adiposity indexes has been also reported in most [12]–[16], but not all [17], cross-sectional assessments. Some clinical trials have also added support to this association [18]–[20].

All these studies have mainly used full-length food frequency questionnaires (FFQ) usually with >100 items, 24-hour recalls or other time-consuming methods, to evaluate the adherence to the Mediterranean dietary pattern. In contrast with these methods, a brief tool assessing only a small number of foods measured in servings/d or servings/wk might enable a dietitian to provide immediate feedback to participants and to establish negociated changes with them to improve their dietary quality, setting the goals in easily understandable units (food servings) [21]. The available studies in adults assessed body weight, weight changes or changes in body mass index (BMI) as measures of adiposity. Nevertheless, it is well known that measures of abdominal obesity outperform body weight or BMI in predicting cardiovascular risk or diabetes [22]–[25]. Waist circumference (WC) has been most commonly used to assess abdominal obesity. However, WC does not take differences in height into account. It is very likely that subjects with a given WC will have more abdominal fat than taller subjects with the same WC. The use of the waist-to-height ratio (WHtR) instead of WC overcomes this problem by taking height into account. The use of the WHtR is supported by a systematic review which assessed WHtR, WC, and BMI as predictors of diabetes or cardiovascular disease and found mean areas under receiving operator characteristic curves of 0.704, 0.693 and 0.671 for WHtR, WC and BMI, respectively [23]. A prior meta-analysis [24] and a study specifically examining this issue in Mediterranean subjects [25] also supported the predictive superiority of WHtR.

To our knowledge, with the exception of a Spanish study in children [15], no previous study in adults has assessed the association between the overall quality of a diet and WHtR. We assessed the association between adherence to the Mediterranean diet (as measured with a simple 14-item questionnaire [26]) and WHtR or other indexes of adiposity in all the participants at baseline of the PREDIMED study, a large trial of nutritional intervention for the primary prevention of cardiovascular disease [27].

## Methods

The design and methods of the PREDIMED trial have been reported in a specific publication [27]. PREDIMED is a randomized, cardiovascular primary prevention trial conducted in 11 Spanish recruiting centers (www.predimed.es, www.predimed.org). The protocol was approved by the Institutional Review Boards at all study locations. The trial is registered at http://www.controlled-trials.com/ISRCTN 35739639. The PREDIMED trial has included 7,447 participants who were randomly allocated to one of three arms: 1) a traditional Mediterranean diet supplemented with extra virgin olive oil; 2) a traditional Mediterranean diet supplemented with tree nuts; or 3) a control (low-fat) diet. The primary cardiovascular composite end-point includes either non-fatal acute myocardial infarction, non-fatal stroke, or cardiovascular death. The final results regarding the primary end-point are expected for 2012/13. All data used in the present report were collected at the beginning of the trial and correspond, thus, only to the baseline evaluations.

### Subjects

Participants were women (60 to 80 years) or men (55 to 80 years) without prior cardiovascular disease, but at high cardiovascular risk because they had either type-2 diabetes mellitus or at least three major cardiovascular risk factors out of six candidate risk factors. These risk factors were: current smoking, hypertension, elevated low-density lipoprotein cholesterol, low high-density lipoprotein cholesterol, overweight/obesity, or family history of premature coronary heart disease. The specific cut-off points for these factors and the exclusion criteria have been previously described [27]. The methods for the dietary intervention of the PREDIMED trial have been also published [21], [27]. Participants can be assumed to be on stable weights at the time of recruitment for the trial. Energy restriction was not part of the PREDIMED nutritional intervention.

Participants were recruited between 2003 and 2009 in Spanish Primary Care Centers affiliated to the 11 recruiting centers. Eighty-nine percent of candidate subjects who met entry requirements agreed to participate and provided written informed consent.

### Measurements

At baseline, registered dietitians completed a 14-item Mediterranean Diet adherence screener (Table 1) in a face-to-face interview with the participant [21], [27]–[29]. The dietitians had been previously trained and certified to implement the PREDIMED protocol and had been hired to work full-time for the trial. The 14-item tool was developed in a Spanish case-control study of myocardial infarction [30], where the best cut-off points for discriminating between cases and controls were selected for each food or food group. With this first step, 9 of the 14 items were obtained [31]. Five additional items that were felt to be especially relevant to assess adherence to the traditional Mediterranean diet were subsequently added. Two of these items used short questions to inquire on food habits: *Do you use olive oil as the principal source of fat for cooking?* and *Do you prefer to eat chicken, turkey or rabbit instead of beef, pork, hamburgers or sausages?* The other 3 items inquired on frequency of consumption of nuts, soda drinks and a typical Mediterranean sauce (“sofrito”): *How many times do you consume nuts per week? How many carbonated and/or sugar-sweetened beverages do you consume per day? How many times per week do you consume boiled vegetables, pasta, rice, or other dishes with a sauce (“sofrito”) of tomato, garlic, onion, or leeks sauteed in olive oil?* [26].

**Table 1:** Validated 14-item Questionnaire of Mediterranean diet adherence.

Questions	Criteria for 1 point
1. Do you use olive oil as main culinary fat?	Yes
2. How much olive oil do you consume in a given day (including oil used for frying, salads, out-of-house meals, etc.)?	≥4 tbsp
3. How many vegetable servings do you consume per day? (1 serving : 200 g [consider side dishes as half a serving])	≥2 (≥1 portion raw or as a salad)
4. How many fruit units (including natural fruit juices) do you consume per day?	≥3
5. How many servings of red meat, hamburger, or meat products (ham, sausage, etc.) do you consume per day? (1 serving: 100–150 g)	<1
6. How many servings of butter, margarine, or cream do you consume per day? (1 serving: 12 g)	<1
7. How many sweet or carbonated beverages do you drink per day?	<1
8. How much wine do you drink per week?	≥7 glasses
9. How many servings of legumes do you consume per week? (1 serving : 150 g)	≥3
10. How many servings of fish or shellfish do you consume per week? (1 serving 100–150 g of fish or 4–5 units or 200 g of shellfish)	≥3
11. How many times per week do you consume commercial sweets or pastries (not homemade), such as cakes, cookies, biscuits, orcustard?	<3
12. How many servings of nuts (including peanuts) do you consume per week? (1 serving 30 g)	≥3
13. Do you preferentially consume chicken, turkey, or rabbit meat instead of veal, pork, hamburger, or sausage?	Yes
14. How many times per week do you consume vegetables, pasta, rice, or other dishes seasoned with sofrito (sauce made with tomatoand onion, leek, or garlic and simmered with olive oil)?	≥2

The baseline 14-item questionnaire (Table 1) was the primary measure used in this study to appraise adherence of participants to the Mediterranean diet. In addition, a full-length 137-item validated FFQ [32] was also administered to all participants. We obtained information about total energy intake and alcohol intake (only with descriptive purposes) from this FFQ. In the validation study, the score obtained with brief 14-item questionnaire correlated significantly with that obtained from the full-length FFQ score (Pearson correlation coefficient (r) = 0.52; intraclass correlation coefficient = 0.51). Associations in the anticipated directions for the different dietary intakes reported on the FFQ were found [26]. Significant inverse correlations of the 14-item tool with fasting glucose, total:HDL cholesterol ratio, triglycerides and the 10-y estimated coronary artery disease risk also supported the validity of this brief Mediterranean diet adherence screener [26].

Also a general medical questionnaire, and the validated Spanish version of the Minnesota Leisure-Time Physical Activity Questionnaire [33]–[34] were collected by the dietitians in the personal interview with each participant [21]. Weight, height and WC were directly measured by registered nurses who had been previously trained and certified to implement the PREDIMED protocol and were hired to work full-time for this trial, as previously described [21], [27]–[29]. The WHtR was calculated as WC divided by height, both in centimeters.

### Statistical Analyses

We compared the baseline characteristics of participants according to three categories of adherence to the Mediterranean diet (≤5, 6–9 and ≥10 points of the 14-item questionnaire). We calculated means (SD) or percentages for each variable across the three categories and assessed the statistical significance of the differences among them with one-way ANOVA and chi-squared tests, respectively.

We compared means and 95% confidence intervals (CI) of the three adiposity indexes (BMI, WC and WHtR) across 5 categories of the 14-item score of adherence to the Mediterranean diet (≤5, 6–7, 8, 9, ≥10 points). Comparisons were done separately for women and men. We also calculated the Pearson correlation coefficients (r) between each of the three adiposity indexes and the 14-item score. Because the Pearson coefficient is sensitive to data distribution, we also calculated Spearman rank correlations. We also estimated partial correlations after accounting for total energy intake.

We used multivariable linear regression modelling to compare adjusted differences in the three adiposity indexes between participants with the lowest adherence to the Mediterranean diet (score ≤7, reference category) and those with higher levels of adherence (8–9, or ≥10). We also used the 14-item score as a continuous variable and assessed differences in each of the three indexes for a two-point increment. Comparisons were done separately for women and men and were adjusted for age (continuous), smoking (3 categories), diabetes status, hypertensive status, physical activity (METS-h/day, continuous), educational level (3 categories), marital status, and centre. Additionally, we also adjusted for total energy intake. We also used multivariable logistic regression to estimate odds ratios (OR) for obesity (BMI≥30 kg/m^2^) or abdominal obesity (defined as WHtR>0.6) for participants with higher levels of adherence to the Mediterranean diet (8–9, or ≥10 points in the 14-item score) versus those with lower adherence (≤7 points, reference category) in separated models for women and men and after adjusting for age, smoking and centre. In another model, we additionally adjusted for diabetes status, hypertensive status, educational level, marital status and physical activity. Finally, we also used multivariable logistic regression to estimate the association between each of the 14 individual items of the score and the odds of obesity (BMI≥30 kg/m^2^) or abdominal obesity (WHtR>0.6) after adjusting for sex, age, smoking and centre. Additionally, we also adjusted for all the other items in the score. A p value <0.05 was considered as statistically significant. Stata 12.0 was used for all analyses.

## Results

We included all 7,447 participants (4,282 women and 3,165 men) in the PREDIMED trial. The mean (± SD) of the 14-item score was 8.6±2.0. Mean values (± SD) for the 14-item score were 8.5±2.0 for women and 8.7±2.0 for men. More men than women were at or above 10 points in the 14-item score (Table 2). No differences in average age, prevalence of hypertension or prevalence of dyslipidemia were found across categories of adherence to the Mediterranean diet. The prevalence of diabetes and current smoking were lower among participants with higher adherence to the Mediterranean diet, whereas the prevalence of family history of coronary heart disease was higher in participants scoring ≥10. Adherence to the Mediterranean diet was directly associated with physical activity, total energy intake, alcohol consumption, and educational level. Married subjects exhibited a greater adherence to the Mediterranean diet than did subjects in other categories of marital status.

**Table 2:** Characteristics of participants by adherence to the Mediterranean diet (14-item score).

	Women				Men			
Adherence to MeDiet	≤5	6–9	≥10		≤5	6–9	≥10	
*n*	264	2584	1434	p*	176	1837	1152	p*
Age, yr	68±5.6	68±5.8	67±5.9	0.058	66±6.8	66±6.6	66±6.5	0.90
CHD family history, %	21	26	28	0.043	16	16	19	0.101
Hypertensión, %	88	87	86	0.58	79	79	76	0.30
Dyslipidemia, %	80	76	78	0.056	67	67	65	0.78
Diabetes, %	47	47	41	0.001	55	56	50	0.010
Smoking								
Current smokers, %	8	6	5	0.016	32	26	23	0.076
Former smokers, %	6	6	9		45	47	50	
Phys. activity (METS-min/d)	2.4±2.4	2.7±2.7	3.1±2.9	<0.001	4.5±4.4	4.9±4.6	5.7±5.3	<0.001
Total energy intake, kcal/d	2103±555	2100±562	2240±544	<0.001	2528±668	2397±629	2512±606	<0.001
Alcohol intake, g/d	2.1±4.6	2.8±5.8	4.1±7.0	<0.001	11.4±14.0	14.6±18.9	17.4±17.5	<0.001
Marital status								
Single, %	5	4	4	0.16	6	4	5	0.21
Married, %	67	65	70		86	90	90	
Widowed, %	25	27	23		3	4	3	
Educational level								
Primary or less, %	89	87	83	0.002	71	68	66	0.25
Secondary, %	7	10	12		22	21	22	
Higher, %	4	3	5		7	12	12	

Means ± SD unless otherwise stated.

* One-way ANOVA tests (continuous variables) or chi squared tests (categorical variables).

For all the three indexes of obesity, we found an apparent inverse association with the 14-item Mediterranean diet score both for women and men in the crude analyses (p<0.001 for all comparisons, with the exception of p = 0.001 for BMI among men) (Table 3). However, the correlation coefficient was strongest for the WHtR, both among women and men (Table 3). Specifically, the inverse correlation coefficient between the 14-point score and WHtR (r = –0.121 for men and women considered together) was greater than the inverse correlation for WC (r = –0.095) or BMI (r = –0.080). This was also true when the non-parametric Spearmańs rho was used (rho = –0.124 for WHtR, rho = –0.097 for WC and rho = –0.092 for BMI). Partial correlations controlling for total energy intake between the 14-point score and adiposity indexes rendered similar results with r_partial_ = –0.118 for WHtR, –0.101 for WC and –0.084 for BMI.

**Table 3:** Means (95% confidence intervals) for Indexes of general obesity and abdominal obesity by adherence to the Mediterranean diet.

	Adherence to Mediterranean diet (0 to 14 point score)							
	≤5	6–7	8	9	≥10	r(Pearson*)	p**	
**Women (n)**	**(264)**	**(938)**	**(826)**	**(820)**	**(1434)**			
Body mass index (kg/m^2^)	31.34 (27.54–35.14)	30.68 (28.72–32.65)	30.61 (28.52–32.70)	30.36 (28.28–32.44)	30.05 (28.49–31.61)	−0.084	<0.001	
Waist circumference (cm)	101.7 (88.9–114.5)	99.4 (92.9–105.8)	99.1 (92.2–106.0)	98.0 (91.2–104.9)	96.9 (91.8–102.0)	−0.125	<0.001	
Waist to height ratio	0.66 (0.58–0.74)	0.64 (0.60–0.69)	0.64 (0.60–0.69)	0.64 (0.59–0.68)	0.63 (0.59–0.66)	−0.132	<0.001	
**Men (n)**	**(176)**	**(626)**	**(604)**	**(607)**	**(1152)**			
Body mass index (kg/m^2^)	29.98 (25.52–34.44)	29.49 (27.18–31.81)	29.49 (27.13–31.84)	29.31 (26.97–31.65)	29.08 (27.40–30.76)	−0.058	0.001	
Waist circumference (cm)	105.0 (88.9–121.0)	104.1 (95.8–112.4)	104.2 (95.7–112.7)	103.1 (94.8–111.5)	102.0 (96.1–108.0)	−0.087	<0.001	
Waist to height ratio	0.63 (0.54–0.72)	0.62 (0.57–0.67)	0.62 (0.57–0.67)	0.62 (0.57–0.67)	0.61 (0.58–0.65)	−0.089	<0.001	

* The corresponding values using the Spearman rank correlation coefficients were among women: −0.099 (body mass index), −0.123 (waist), and −0.132 (waist-to-height). The respective Spearman correlations for men were: −0.068, −0.099 and −0.099.

** Crude linear regression model.

The inverse association between the score of adherence to the Mediterranean diet and the three indexes of obesity remained statistically significant in multivariable analyses (Table 4). For each 2 additional points in the 14-item Mediterranean diet score, the WHtR was 0.0066 lower (95% CI, –0.0088 to –0.0049) in women and 0.0059 lower (–0.0079 to –0.0038) in men. These estimates did not appreciably change after adjustment for total energy intake obtained from the FFQ.

**Table 4:** Multivariable-adjusted differences (95% confidence intervals) in indexes of general obesity and abdominal obesity by adherence to the Mediterranean diet.

	Adherence to the Mediterranean diet (0 to 14 point score)^1^					
	≤7 (ref)	8–9	≥10		For +2 points^1^	For +2 points^2^
**Women (n)**	**(1202)**	**(1646)**	**(1434)**			
Body mass index (kg/m^2^)	0 (ref.)	−0.37 (−0.66 to −0.07)	−0.76 (−1.08 to −0.49)		−0.39 (−0.52 to −0.26)	−0.37 (−0.50 to −0.24)
Waist circumference (cm)	0 (ref.)	−0.73 (−1.51 to +0.05)	−1.78 (−2.60 to −0.96)		−0.92 (−1.26 to −0.59)	−0.92 (−1.26 to −0.58)
% Waist to height ratio^3^	0 (ref.)	−0.57 (−1.06 to −0.08)	−1.32 (−1.84 to −0.80)		−0.66 (−0.88 to −0.49)	−0.65 (−0.87 to −0.48)
**Men (n)**	**(802)**	**(1211)**	**(1152)**			
Body mass index (kg/m^2^)	0 (ref.)	−0.26 (−0.55 to +0.04)	−0.61 (−0.92 to −0.31)		−0.25 (−0.37 to −0.12)	−0.25 (−0.37 to −0.13)
Waist circumference (cm)	0 (ref.)	−0.92 (−1.79 to −0.06)	−2.36 (−3.25 to −1.47)		−0.95 (−1.30 to −0.59)	−0.93 (−1.29 to −0.58)
% Waist to height ratio^3^	0 (ref.)	−0.62 (−1.11 to −0.12)	−1.40 (−1.92 to −0.89)		−0.59 (−0.79 to −0.38)	−0.58 (−0.79 to −0.38)

1 Adjusted for age (continuous), smoking (3 categories), diabetes status (dichotomous), hypertensive status (dichotomous), physical activity (continuous), educational level (3 categories), marital status (4 categories), and centre (11 centres).

2 Additionally adjusted for total energy intake (continuous).

3 A Waist-to-height ratio = 1 is taken as 100%.

We also found an inverse association between adherence to the Mediterranean diet and both general obesity and abdominal obesity. The OR of abdominal obesity (WHtR>0.6) for each 2 additional points in the score, was 0.85 among women (95% CI, 0.79 to 0.92) and 0.84 among men (0.88 to 0.91) in fully-adjusted models (Table 5).

**Table 5:** Prevalence of obesity and multivariable-adjusted odds ratios (OR, 95% confidence intervals) for abdominal obesity and general obesity by adherence to the Mediterranean diet.

	Adherence to the Mediterranean diet			
	≤7 (ref)	8–9	≥10	For +2 points
**Women (n)**	**(1202)**	**(1646)**	**(1434)**	
Abdominal obesity^1^ (%. unadjusted)	76.8	73.0	66.0	
Adjusted OR^2^	1 (ref.)	0.86 (0.72–1.03)	0.63 (0.53–0.76)	0.81 (0.75–0.87)
Additionally adjusted^3^	1 (ref.)	0.88 (0.74–1.06)	0.70 (0.58–0.84)	0.85 (0.79–0.92)
Obesity^4^ (%, unadjusted)	56.0	53.8	46.3	
Adjusted OR^2^	1 (ref.)	0.89 (0.76–1.03)	0.63 (0.54–0.74)	0.83 (0.78–0.88)
Additionally adjusted^3^	1 (ref.)	0.89 (0.76–1.05)	0.68 (0.57–0.80)	0.82 (0.77–0.88)
**Men (n)**	**(802)**	**(1211)**	**(1152)**	
Abdominal obesity^1^ (%. unadjusted)	66.7	64.7	57.3	
Adjusted OR^2^	1 (ref.)	0.87 (0.72–1.06)	0.63 (0.52–0.76)	0.84 (0.77–0.90)
Additionally adjusted^3^	1 (ref.)	0.87 (0.71–1.05)	0.65 (0.53–0.79)	0.84 (0.78–0.91)
Obesity^4^ (%. unadjusted)	46.3	40.1	36.7	
Adjusted OR^2^	1 (ref.)	0.76 (0.63–0.91)	0.62 (0.52–0.76)	0.83 (0.77–0.90)
Additionally adjusted^3^	1 (ref.)	0.76 (0.63–0.92)	0.66 (0.54–0.80)	0.84 (0.78–0.91)

1 Waist to height ratio ≥0.6.

2 Adjusted for age (continuous), smoking (3 categories) and centre (11 centres).

3 Additionally adjusted for diabetes status (dichotomous), hypertensive status (dichotomous), educational level (3 categories), marital status (4 categories) and physical activity (continuous).

4 Body mas index ≥30 kg/m^2^.

**Figure 1:**
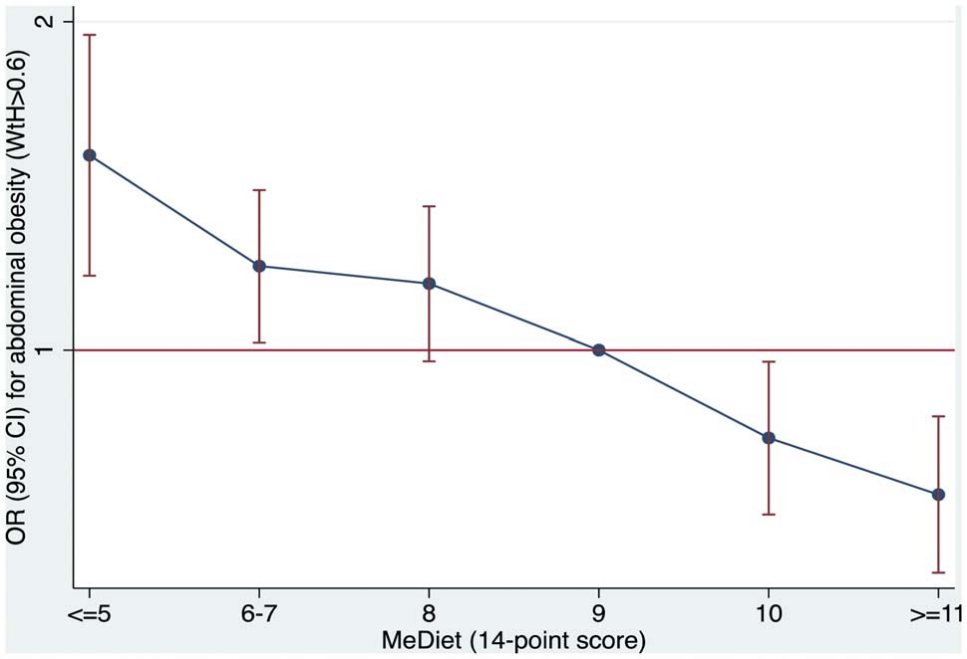
Multivariable-adjusted odds ratios (OR, 95% confidence intervals) for abdominal obesity (waist-to-height>0.6) by adherence to the Mediterranean diet. Adjusted for sex, age, smoking and centre.

When both men and women were considered together, a monotonic inverse association between the 14-item score and the odds of abdominal obesity (WHtR>0.6) was apparent (Figure 1). Taking as reference 9 points (OR = 1.00), significantly lower odds for abdominal obesity (WHtR>0.6) were found for participants scoring either 10 points (OR 0.83; CI 0.71 to 0.98) or ≥11 points (OR 0.74; 0.62 to 0.87), whereas significantly higher odds were found for those scoring 6–7 points (OR 1.19; 1.02 to 1.40), or ≤5 points (OR 1.51; 1.17 to 1.94).

**Table 6:** Multivariable-adjusted^1^ odds ratios (OR, 95% confidence intervals) for abdominal obesity and general obesity according to the fulfilment of each item included in the 14-point screener of adherence to the Mediterranean diet.

	% positive at recruitment	OR for Abdominal Obesity^2^ (95% CI)	P	OR for Obesity^3^ (95% CI)	P
**1. Use of olive oil as main culinary lipid**	89.8	0.85 (0.71–1.01)	0.061	0.84 (0.72–0.99)	0.032
**2. Olive oil >4 tablespoons**	70.0	0.95 (0.84–1.06)	0.349	0.99 (0.89–1.10)	0.850
**3. Vegetables ≥2 servings/d**	42.1	0.80 (0.72–0.89)	<0.001	0.83 (0.75–0.91)	<0.001
**4. Fruits ≥3 servings/d**	51.3	0.85 (0.77–0.94)	0.002	0.94 (0.86–1.04)	0.232
**5. Red/processed meats <1/d**	86.9	0.90 (0.77–1.05)	0.177	0.70 (0.61–0.81)	<0.001
**6. Butter. cream. margarine <1/d**	89.9	1.07 (0.91–1.27)	0.414	1.19 (1.02–1.39)	0.031
**7. Soda drinks <1/d**	88.7	0.78 (0.66–0.92)	0.004	0.85 (0.73–0.98)	0.030
**8. Wine glasses ≥7/wk**	29.5	0.88 (0.79–0.99)	0.040	0.79 (0.71–0.88)	<0.001
**9. Legumes ≥3/wk**	26.8	0.87 (0.77–0.98)	0.021	0.90 (0.81–1.01)	0.067
**10. Fish/seafood ≥3/wk**	56.0	0.87 (0.79–0.97)	0.011	0.90 (0.81–0.99)	0.024
**11. Commercial sweets and confectionery ≤2/wk**	66.9	0.91 (0.82–1.02)	0.104	0.87 (0.79–0.96)	0.006
**12. Tree nuts ≥3/wk**	34.0	0.67 (0.61–0.75)	<0.001	0.65 (0.59–0.72)	<0.001
**13. Poultry more than red meats**	66.7	0.97 (0.87–1.08)	0.572	0.91 (0.82–1.01)	0.083
**14. Use of sofrito sauce ≥2/wk**	62.9	0.87 (0.78–0.97)	0.016	0.90 (0.81–1.00)	0.048

1 Adjusted for sex, age (continuous), smoking (3 categories), and centre (11 centres),

2 Waist to height ratio **≥**0.6.

3 Body mas index **≥**30 kg/m^2^.

With the exception of one item (butter/cream/margarine), all the odds ratios for the individual items included in the 14-item Mediterranean diet score showed point estimates suggesting inverse associations with the odds of abdominal obesity (Table 6). Changes in these point estimates before and after additional adjustments for other potential confounders were small. In any case, the results were not statistically significant for four items: the second of the two items for olive oil consumption (p = 0.35), low consumption of red/processed meats (p = 0.18), low use of commercial bakery (p = 0.10) and preference for poultry instead of red meats (p = 0.57). The strongest inverse association was found for the consumption of nuts (OR 0.67; 0.61 to 0.75, p<0.001). With slight differences for some items, the results were basically the same when we estimated the OR for general obesity (Table 6). Contrary to expectations, we found a significantly direct, instead of inverse, association of the item combining margarine, butter and cream with obesity and average BMI, i.e., participants with a higher consumption of margarine, butter or cream exhibited a *lower* BMI (adjusted difference: 0.44 kg/m^2^; CI, 0.15 to 0.73, after adjusting for potential confounders and all the other items in the score). Participants answering ≥1 to the question *How many servings of butter, margarine, or cream do you consume per day? (1 serving: 12 g)* had mainly a higher consumption of margarine (6 g/d), with minor consumptions of butter (2.6 g/d) or cream (0.6 g/d). Therefore, this item was mainly related to margarine consumption. When we used the FFQ to assess the association between consuming ≥1 serving/d of margarine, an inverse association with obesity was found with OR = 0.84 (95% CI: 0.68–1.03, p = 0.09) after adjusting for age, sex, centre and smoking. This inverse association between margarine and adiposity was weaker and statistically non-significant for abdominal obesity (WHtR>0.6) with OR = 0.93 (0.74–1.17). No association was observed for butter or cream.

**Figure 2:**
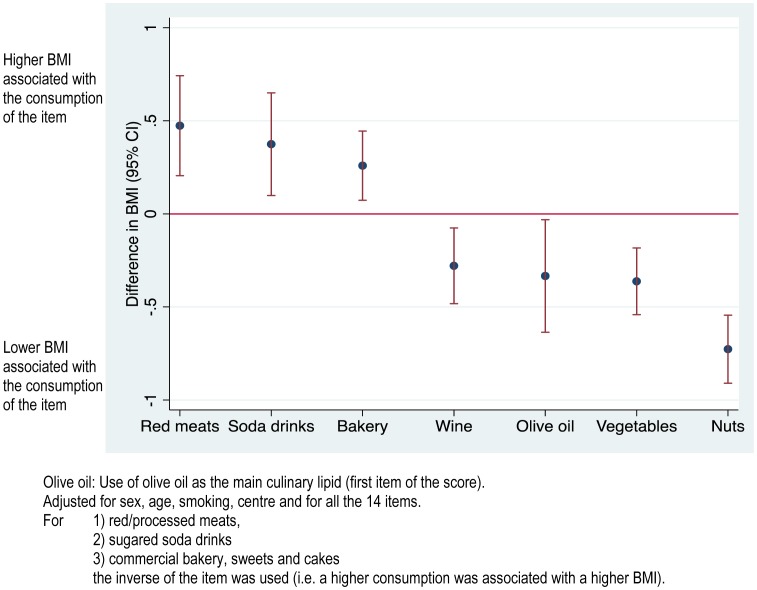
Adjusted differences in BMI for 7 selected items in the 14-point score of adherence to the Mediterranean diet independently associated with BMI. Olive oil: Use of olive oil as the main culinary lipid (first item of the score). Adjusted for sex, age, smoking, centre and for all the 14 items. For 1) red/processed meats, 2) sugared soda drinks, 3) commercial bakery, sweets and cakes the inverse of the item was used (i.e. a higher consumption was associated with a higher BMI).

**Figure 3:**
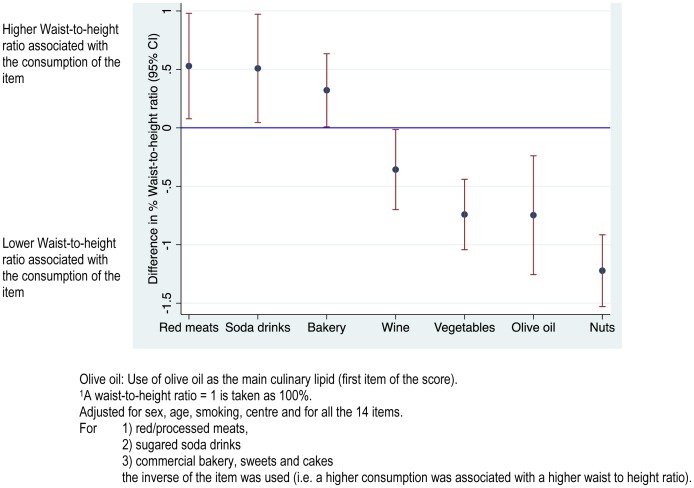
Adjusted differences in % waist-to-height ratio (95% confidence intervals)^1^ for selected items in the 14-point score of adherence to the Mediterranean diet independently associated with the waist-to-height ratio. Olive oil: Use of olive oil as the main culinary lipid (first item of the score). ^1^A waist-to-height ratio = 1 is taken as 100%. Adjusted for sex, age, smoking, centre and for all the 14 items. For 1) red/processed meats, 2) sugared soda drinks, 3) commercial bakery, sweets and cakes, the inverse of the item was used (i.e. a higher consumption was associated with a higher waist to height ratio).

When we introduced separately all the 14 items in a model with BMI as the dependent variable, also adjusted for age, sex, smoking and center, we found that higher consumptions of red meat, soda drinks, and commercial bakery/sweets/cakes were significantly associated with higher average BMI, whereas higher consumption of wine, use of olive oil as the main culinary lipid, vegetables, and nuts were associated with lower average BMI (Figure 2). High fruit consumption did not show a significant independent association with BMI (p = 0.76). Similar associations were found when we used WHtR instead of BMI as the dependent variable (Figure 3). In this analysis the association between high fruit consumption and lower WHtR approached the limit of statistical significance (p = 0.07).

## Discussion

In this baseline assessment of 7,447 participants in the PREDIMED primary prevention trial we found a robust and monotonic inverse association between adherence to the Mediterranean diet and indexes of abdominal obesity or general obesity. This is the first time that an inverse association between adherence to the Mediterranean diet and the WHtR has been reported in an adult population using an easily administrable brief tool to assess the overall quality of diet and its conformity to the traditional Mediterranean dietary pattern. Prior findings on the relationship between the Mediterranean diet and WC based on full-length dietary assessment methods are consistent with this finding [14], [20]. The most interesting aspect of this study is that adherence to the Mediterranean diet was appraised using only a brief 14-item questionnaire which is less time-demanding, less expensive and requires less collaboration from participants than the usual full-length FFQ or other more comprehensive methods. In addition, it provides the unique window of opportunity to provide feedback to the participant immediately after the questionnaire is completed. In fact, this 14-item tool is a key element in the intervention conducted in the PREDIMED trial and has been previously validated against the FFQ used in the study [21], [26]. The present results reinforce the usefulness of the 14-point Mediterranean diet screener and its potential application in other settings.

Another contribution of the present work is the use of a novel index for central obesity (WHtR) which is easily measurable, can advantageously replace the use of BMI in the assessment of diet-adiposity relationships and may perform differently to BMI.

Previously we had assessed the first 3204 trial participants and found that the prevalence of diabetes, hypertension and obesity was lower in participants with higher values of the 14-item screener [35]. We have reported also that the 14-item score related directly to HDL-cholesterol and inversely to BMI, WC, serum triglycerides, and fasting glucose among 7146 participants in the PREDIMED trial [28]. In the present study we include all 7447 participants and have assessed in detail not only BMI and WC, but also the WHtR. Importantly, we also report the specific association of each of the 14 individual items included in the Mediterranean diet score with indexes of obesity or abdominal obesity.

Our results suggest that closer adherence to a Mediterranean diet is associated with a lower prevalence of obesity, and specifically, abdominal obesity. This is highly plausible because Mediterranean diets have been shown in rigorously controlled randomized trials [18]–[20], [36]–[37] to provide beneficial metabolic effects, including improvements in insulin sensitivity and reductions in biomarkers of low-grade inflammation [38]–[40]. The excellent nutritional adecuacy that can be obtained by following a Mediterranean diet [41] might add biological plausibility to an inverse association of this dietary pattern with abdominal and visceral adiposity. The appropriate harmony and balance in the intake of both macronutrients and essential micronutrients [42] may contribute to lower oxidative stress, less postprandial inflammation and reductions in other mechanisms associated with the abdominal deposition of fat [43], [44].

Our results are also consistent with previous epidemiologic studies showing a beneficial effect of the Mediterranean diet on the risk of the metabolic syndrome [36]. In addition to the observed stronger inverse association of the Mediterranean diet with the WHtR than with BMI or WC, when we individually assessed each of the 14 items included in the score, almost all of them were associated with a lower prevalence of abdominal obesity. The only exception was the food group that included butter, margarine, and cream. Participants consuming ≥1 serving/d were mainly margarine consumers. A borderline significant (p = 0.09) *inverse* association between margarine consumption (≥1 serving/d) and obesity risk was found. Currently, most margarine brands in Spain do not contain *trans* fatty acids –related to a higher risk of weight gain and abdominal obesity [45], [46]- and there are no reasons to expect that margarine consumption might be linked to a greater obesity risk.

The main limitation of this study relates to the cross-sectional nature of our analyses and the potentiality for reverse causality bias. However, the most likely direction of that potential bias would be towards finding a *higher* adherence to a healthier Mediterranean diet among overweight or obese subjects. This possibility perhaps might explain why a non-significant direct association was reported in a previous cross-sectional assessment [17]. Indeed, is it highly plausible that subjects who perceive themselves as overweight/obese would be more likely to adopt favourable changes in their diets, because the perception of their excess weight would lead them to improve their dietary habits or to receive this advice from their caregivers. Therefore, given this potential bias, we would have expected a *positive* association between adherence to the Mediterranean diet and adiposity indexes, because high-risk patients with overweight/obesity might be more likely to change their food habits as a consequence of their excess weight. Presently, in our setting, some physicians or dietitians would recommend a Mediterranean-type diet to overweight or obese patients. Consequently, the inverse association found in this assessment is unlikely to be due to reverse causation bias. Another potential limitation is the small absolute magnitude of observed differences in the adiposity indexes; also other factors that we did not collect, such as dieting before the trial could represent alternative explanations of our findings.

The final results of the PREDIMED trial will include the prospective assessment of weight, WC and WHtR changes after an average follow-up of 5 years according to the randomized intervention with an intention to treat analysis. These results will be available in the near future and should provide a definitive confirmation of our findings.
